# Runx1-Snx9 axis drives the pathological secretion of mitochondrial-derived vesicles to activate cGAS-STING signaling in acute pancreatitis

**DOI:** 10.1186/s12951-026-04687-6

**Published:** 2026-06-16

**Authors:** Mengqi Gao, Guohui Xiao, Kunhao Chen, Shiyu Li, Cong Chen, Sifei Yu, Yahui Wu, Kaige Yang, Rongli Xie, Erzhen Chen, Jian Jiang, Ying Chen, Jian Fei, Enqiang Mao, Dan Xu

**Affiliations:** 1 Department of Emergency, Ruijin Hospital, Shanghai Jiao Tong University School of Medicine, Shanghai, 200025 China; 2 Department of General Surgery, Pancreatic Disease Center, Ruijin Hospital, Shanghai Jiao Tong University School of Medicine, Shanghai, 200025 China; 3 Department of General Surgery, Ruijin Hospital LuWan Branch, Shanghai Jiao Tong University, Shanghai, 200020 China; 4 Shanghai Institute of Aviation Medicine, Ruijin Hospital, Shanghai Jiao Tong University School of Medicine, Shanghai, 200025 China; 5 Department of Immunology & Institute of Geriatric Immunology, School of Medicine, Jinan University, Guangzhou, 510632 China; 6 Department of Emergency, The First People’s Hospital of Taicang, Taicang Affiliated Hospital of Soochow University, No. 58, Changsheng South Road, Taicang City, Suzhou City, Jiangsu, 215400 China

**Keywords:** Acute Pancreatitis, Runx1, Snx9, MDV, cGAS-STING signaling

## Abstract

In acute pancreatitis (AP), the release of mitochondrial DNA (mtDNA) from pancreatic acinar cells (PACs) plays a pivotal role in triggering a lethal systemic inflammatory response. Despite the importance of mtDNA release, the regulatory mechanisms upstream of this event remain poorly understood, hindering the development of targeted therapeutic strategies. To address this, we utilized single-cell RNA sequencing, CUT&Tag, luciferase reporter assays, and experiments in a PAC-specific knockout mouse model to investigate the transcriptional program governing vesicle transport and mtDNA release in the context of AP. Our analysis revealed that vesicle transport pathways were activated in AP PACs and identified Runx1 as a core transcriptional regulator. We discovered that Runx1 directly binds and activates the Snx9 promoter. This interaction initiates a pathological cascade wherein Runx1-Snx9 signaling drives mitochondrial fragmentation and the biogenesis of intracellular mitochondrial-derived vesicles (MDVs). Under AP conditions, these MDVs are diverted from degradative pathways and routed to the secretory machinery to be released as pathogenic, extracellular mitochondrial-derived vesicles (Ex-MDVs). These Ex-MDVs were confirmed to be highly pathogenic, strongly activating the cGAS-STING pathway in macrophages. Notably, PAC-specific deletion of Runx1 in a mouse model significantly mitigated pancreatic injury and suppressed the systemic inflammatory storm associated with AP. This study is the first to elucidate the Runx1-Snx9 transcriptional axis as the core upstream mechanism responsible for the anomalous generation and secretion of Ex-MDVs from PACs during AP, providing novel insights into AP pathogenesis and identifying this axis as a potential therapeutic target.

**Graphical abstract:**

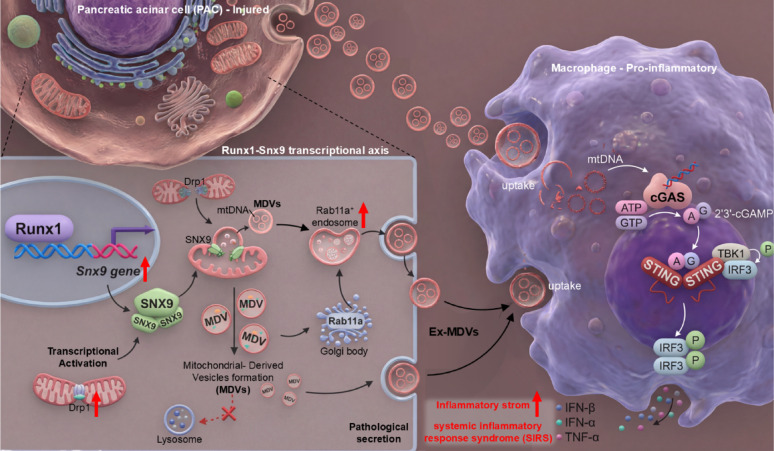

**Supplementary Information:**

The online version contains supplementary material available at 10.1186/s12951-026-04687-6.

## Introduction

Severe acute pancreatitis (AP) is a highly lethal inflammatory disease characterized by persistent organ failure, with mortality rates reaching 20–30% in complicated cases [1]. Its clinical severity is driven not only by local pancreatic injury but also by a systemic inflammatory storm that rapidly escalates beyond the pancreas [2]. Recent studies have identified extracellular mitochondrial DNA (mtDNA) as a key danger-associated molecular pattern (DAMP) that triggers this response [3]. Elevated circulating mtDNA levels are strongly associated with AP disease severity and adverse clinical outcomes [4], supporting the hypothesis that mtDNA serves as a critical molecular link bridging local acinar damage to systemic pathology.

Once released from damaged cells, mtDNA functions as a potent DAMP that orchestrates the inflammatory storm characteristic of AP [5]. Its pathological effects are mediated through the activation of multiple innate immune pathways. Extracellularly, mtDNA is a well-established ligand of Toll-like receptor 9 (TLR9) [6], whereas its presence in the cytoplasm can engage the NLRP3 inflammasome [7] and, critically, the cyclic GMP-AMP synthase (cGAS)-stimulator of interferon genes (STING) signaling axis [8]. The collective activation of these sensors culminates in the massive production of proinflammatory cytokines and type I interferons, which propagate pancreatic injury and drive systemic organ failure [1]. Therefore, elucidating the precise mechanisms governing the initial release of mtDNA is of paramount importance, as it represents the apex of this devastating inflammatory cascade.

Although the pathogenic significance of mtDNA is well established, the precise mechanism of its release into the extracellular space remains unresolved. The prevailing view has historically attributed this event to passive leakage during necrotic cell death [9]; however, accumulating evidence indicates that cells can also export mtDNA through regulated pathways, particularly via extracellular vesicles (EVs) [10, 11]. Intracellularly, mitochondria maintain homeostasis by shedding damaged components via mitochondrial-derived vesicles (MDVs), a process typically coupled with lysosomal degradation (micromitophagy). However, under specific stress conditions, this quality control machinery can be subverted: MDVs may evade degradation and instead fuse with the endosomal system, leading to their aberrant secretion. While this suggests a sophisticated cargo sorting mechanism, the specific transcriptional programs that hijack this machinery to orchestrate the pathological release of extracellular mitochondrial-derived vesicles (Ex-MDVs) in the context of AP remain poorly understood.

To bridge this knowledge gap, we integrated single-cell RNA sequencing (scRNA-seq) data from AP murine models with functional validation in primary pancreatic acinar cells (PACs) to systematically define the regulators of vesicle trafficking. Our work identifies a previously unrecognized Runx1-Snx9 transcriptional axis as a critical pathway regulating the biogenesis of intracellular MDVs and their subsequent pathological secretion as Ex-MDVs. We demonstrate that this axis facilitates the active transport of mtDNA out of acinar cells, thereby activating cGAS-STING signaling in macrophages and amplifying the systemic inflammatory storm.

## Materials and methods

### AP mouse model

AP Mouse Model All animal experiments were approved by the Shanghai Jiao Tong University Institutional Animal Care and Use Committee (Ethics Approval No. SYXK2018-0027) and conducted in accordance with the Guidelines for the Care and Use of Laboratory Animals. AP was induced using a standard cerulein hyperstimulation protocol [12]. Briefly, adult male C57BL/6 mice (6–8 weeks old; Shanghai SLAC Laboratory Animal Co., China) were administered weekly intraperitoneal injections of cerulein (100 µg/kg; Sigma–Aldrich, St. Louis, MO, USA) for 10 consecutive hours. This regimen recapitulates the key pathological features of pancreatitis, including acinar necrosis, inflammatory infiltration, and enzyme release. Control mice received equivalent volumes of saline. To assess the specific role of Runx1 in acinar cells, PAC-specific Runx1 knockout mice (*Runx1*^fl/fl^*Pdx1*^cre^) were generated using a Cre-LoxP system. Genotypes were confirmed via PCR. Animals were randomly assigned to experimental groups based on genotype and treatment. The cGAS inhibitor RU.521 (S6841, Selleck) was sequentially dissolved in 2% DMSO and 98% corn oil. The administered by intraperitoneal injection at a dose of 5 mg/kg within 10 min after AP induction.

## Primary PAC isolation and culture

Primary PACs were isolated from the pancreases of *Runx1*^fl/fl^ and *Runx1*^fl/fl^*Pdx1*^cre^ mice as previously described [2]. Cells were cultured in Dulbecco’s modified Eagle’s medium/F12 (DMEM/F12) supplemented with 10% fetal bovine serum. To establish an in vitro model of AP, PACs were exposed to 100 nM cerulein for 24 h. All cell cultures were routinely tested for mycoplasma contamination to ensure experimental integrity.

### Single-cell transcriptomic analysis and subpopulation stratification

Mouse pancreatic tissues were harvested for scRNA-seq. Post-quality control, transcriptomes were obtained from 15,819 single cells (5,732 control; 10,087 AP) [13]. Cells were clustered into 12 distinct populations based on canonical marker expression using Seurat (v4.3.0). To dissect transcriptional heterogeneity within the acinar lineage, Gene Set Variation Analysis (GSVA; v1.42.0) was performed. A curated gene set (“GOBP_VESICLE_TRANSPORT”) was used to calculate pathway activity scores for individual cells. Based on these scores, PACs were stratified into “Trafficking-High” and “Trafficking-Low” subpopulations. This stratification facilitated the identification of upstream transcriptional regulators, including Runx1, driving the vesicle transport phenotype.

### Cleavage under targets and tagmentation (CUT&Tag)

CUT&Tag assays were performed on primary PACs as previously described [14]. Briefly, 1 × 10^5^ PACs were harvested from each sample, permeabilized, and incubated with a primary antibody against Runx1 (Abcam, #92336) overnight at 4 °C. The cells were then incubated with a secondary antibody to tether the protein A–Tn5 transposase fusion (pA-Tn5) to the antibody–chromatin complex. Activated Tn5 simultaneously cleaves and inserts sequencing adaptors adjacent to antibody-bound sites. DNA fragments were purified using a MinElute PCR Purification Kit (Qiagen, Hilden, Germany) and amplified to generate sequencing libraries, which were quantified, pooled, and subjected to paired-end sequencing on an Illumina NovaSeq 6000 platform. Raw reads were trimmed and aligned to the mouse reference genome (mm10) using Bowtie2 (v2.4.4). Peaks were identified using MACS2 (v2.2.7.1) with the default parameters, and reproducibility between replicates was assessed by Pearson correlations. Peak annotation was performed with ChIPseeker, and motif enrichment analysis was conducted using HOMER (v4.11). The functional enrichment of Runx1 target genes was analyzed using the clusterProfiler package (v4.6.0) in R.

### Histology

Pancreatic tissues were fixed in 4% paraformaldehyde, paraffin-embedded, and sectioned at 4 μm. Hematoxylin and eosin (H&E) staining was performed to assess histopathological severity, quantifying acinar necrosis and inflammatory cell infiltration under a light microscope (Olympus, Japan).

### Plasmids, transfection, and luciferase reporter assays

Gain- and loss-of-function studies were conducted using a Runx1 overexpression plasmid (OE-*Runx1*) and shRNA constructs targeting Snx9 (sh-*Snx9*), alongside their respective controls. Transfections were carried out using Lipofectamine 3000 (Invitrogen). For mechanistic validation, fragments of the *Snx9* promoter containing the predicted Runx1 binding site (Wild-Type, WT) or a site-directed mutant sequence (Mut) were cloned into a firefly luciferase reporter vector. PACs were co-transfected with reporter plasmids and OE-*Runx1* or empty vector. Luciferase activity was quantified after 24 h using the Dual-Luciferase^®^ Reporter Assay System (Promega). Data were normalized to Renilla luciferase activity and expressed as fold change over vector control.

### Immunofluorescence assays

To visualize mitochondrial dynamics and intracellular vesicle formation, frozen pancreatic sections or cultured PACs were stained with primary antibodies against TOM20 (mitochondria; 1:200; ab186735) and dsDNA (1:200; ab27156). Secondary detection was performed using Alexa Fluor 488- or 594-conjugated antibodies. High-resolution images were acquired using a Leica confocal microscope to identify mitochondrial fragmentation and extranuclear mtDNA/MDV puncta.

### Flow cytometry

Single-cell suspensions from pancreatic tissues were prepared by enzymatic digestion and filtration through a 70-µm strainer. Cells were blocked with an anti-CD16/32 antibody (BioLegend, USA) and stained with fluorophore-conjugated antibodies against F4/80, CD86, and intracellular inducible nitric oxide synthase (iNOS) (all from BioLegend) at the recommended dilutions. The data were collected on a BD FACSCanto II flow cytometer (BD Biosciences) and analyzed using FlowJo software.

### Ex-MDV isolation and characterization

Ex-MDVs were isolated from PAC culture supernatants via differential ultracentrifugation. Debris was removed by low-speed centrifugation, followed by vesicle pelleting at 100,000 × g (Beckman Coulter Optima XPN-100). The purity and cargo specificity of Ex-MDVs were characterized by Western blot. The profile included positive EV markers (CD9, Alix) and the absence of non-vesicular contaminants (Lamin B1, β-Actin). Notably, Rab11a was analyzed to distinguish the intracellular recycling machinery from secreted vesicle cargo. Vesicle morphology and size distribution (50–150 nm) were validated using Transmission Electron Microscopy (TEM; JEOL Ltd.).

### Macrophage co-culture system

Bone marrow-derived macrophages (BMDMs) were isolated from C57BL/6 mice and differentiated using M-CSF (20 ng/mL) for 7 days. To simulate paracrine signaling, BMDMs were co-cultured with purified PAC-derived Ex-MDVs (10 µg/mL protein equivalent). The culture medium was supplemented with 10% EV-depleted FBS. After 24 h, cGAS–STING pathway activation was assessed by Western blot (cGAS, STING, p-IRF3), and proinflammatory cytokine secretion (IFN-α, IFN-β, TNF-α) was quantified by ELISA.

### Quantitative PCR (qPCR) mtDNA Cargo in Ex-MDVs

To assess the packaging of mitochondrial DNA into vesicles, total DNA was extracted from purified Ex-MDVs (QIAamp DNA Micro Kit). qPCR was performed using primers specific for mitochondrial genes (*mt-Co3*, *mt-ND1*) and the non-coding D-loop (*mt-Dloop*). mtDNA abundance was normalized to total Ex-MDV protein content to determine the mtDNA copy number per microgram of vesicle protein, reflecting the density of DAMP cargo.

### Western blotting

Protein lysates from PACs and Ex-MDVs were analyzed via SDS-PAGE. Blots were probed with antibodies against RUNX1 (ab92336), SNX9 (CST54984), DRP1 (ab184247), CD9 (ab307085), Rab11A (ab316151), Lamin B1 (ab229025), Alix (ab275377), cGAS (ab252416), STING (ab288157), IRF3 (CST4302), P-IRF3 (CST4947), β-Actin (ab8227), and GAPDH (ab181602). HRP-conjugated secondary antibodies (Cell Signaling Technology) and ECL detection reagents (GE Healthcare) were used for visualization. Band intensities were quantified with ImageJ software.

### Mitochondrial ROS measurements

Mitochondrial reactive oxygen species (mtROS) levels in PACs were measured using the MitoSOX™ Red mitochondrial superoxide indicator (Thermo Fisher Scientific). Briefly, PACs were incubated with 5 µM MitoSOX reagent at 37 °C for 10 min in the dark, followed by three washes with prewarmed PBS to remove excess dye. The cells were then fixed in 4% paraformaldehyde for 15 min, permeabilized with 0.1% Triton X-100, and counterstained with DAPI (Beyotime, Shanghai, China) to visualize the nuclei. Fluorescence images were acquired using a Leica confocal laser scanning microscope.

### ELISA

Blood samples from AP-induced and control mice were collected via retro-orbital bleeding and centrifuged at 3,000 × *g* for 10 min at 4 °C to obtain serum. Amylase, lipase, IFN-α, IFN-β, and TNF-α levels in serum and PAC culture supernatants were measured using ELISA kits (R&D Systems, Minneapolis, MN, USA) according to the manufacturer’s instructions. All ELISA measurements were made in triplicate, and the absorbance was recorded using a microplate reader (BioTek, Winooski, VT, USA).

### Statistical analysis

Data are presented as mean ± SD or SEM as indicated. Statistical comparisons were performed using Student’s t-test (two groups) or ANOVA (multiple groups) with GraphPad Prism software. A *p* value < 0.05 was considered statistically significant.

## Results

### Single-cell transcriptomics reveals Runx1-Driven reprogramming of vesicle trafficking pathways in AP acinar cells

**Fig. 1:**
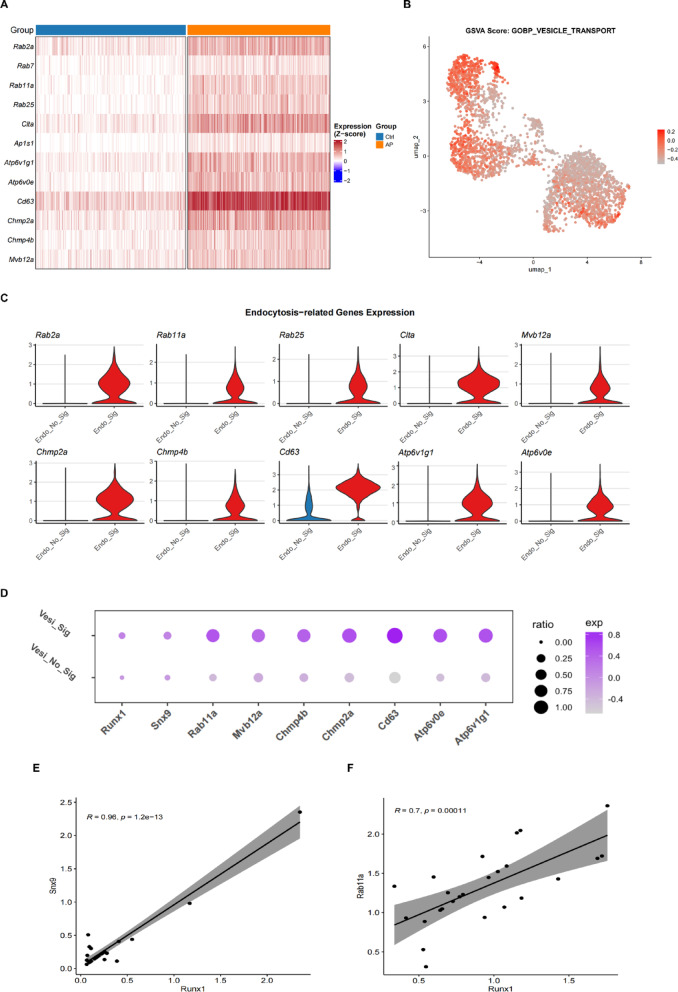
Single-cell transcriptomic analysis reveals Runx1-driven reprogramming of vesicle transport in AP PACs. (**A**) Heatmap of vesicle transport-related gene expression in control and AP PACs. (**B**) UMAP visualization of GSVA scores showing activation of vesicle transport pathways. (**C**) Violin plots of vesicle transport-related genes in control and AP PACs. (**D**) Bubble plot of transcription factor enrichment in the “Trafficking-High” PAC subgroup. (**E**,** F**) Correlation analyses of *Runx1* expression with *Snx9* (**E**) an d *Rab11a* (**F**)

To deconstruct cell-specific molecular architecture of AP, we leveraged single-cell transcriptomic analysis of pancreatic acinar cells (PACs). Unsupervised clustering and heatmap analysis unveiled a profound transcriptional rewiring in AP PACs compared to controls. Specifically, genes governing the vesicular trafficking machinery were globally upregulated in the AP group (Fig. 1A). Crucially, this gene signature was not limited to general transport but was enriched for components critical to alternative secretory pathways, including endosomal recycling (*Rab11a*, *Rab25*) and multivesicular body (MVB) biogenesis (*Mvb12a*, *Chmp4b*). Gene Set Variation Analysis (GSVA) further quantified this pathway activation, demonstrating that AP PACs exhibited significantly higher enrichment scores for vesicle transport pathways compared to homeostatic controls (Fig. 1B). Differential expression analysis identified a specific upregulation of *Snx9*, a BAR-domain protein essential for vesicle scission, alongside *Rab11a* and *Rab2a* (Fig. 1C). The co-upregulation of *Snx9* (implicated in mitochondrial budding) and *Rab11a* (implicated in vesicle exocytosis) suggests that AP induces a coordinated transcriptional program capable of supporting the biogenesis of intracellular MDVs and their subsequent routing to the extracellular space.

To pinpoint the upstream driver of this program, we stratified PACs into “Trafficking-High” (vesicle transport-significant) and “Trafficking-Low” subgroups. Transcription factor enrichment analysis identified Runx1 as a top-ranking regulator specifically enriched in the “Trafficking-High” subset (Fig. 1D). Notably, this Runx1-positive subpopulation was marked by the highest expression of *Snx9*. Pearson correlation analysis revealed a robust positive correlation between *Runx1* and *Snx9* (Fig. 1E), as well as between *Runx1* and *Rab11a* (Fig. 1F). This strong co-expression pattern indicates that Runx1 does not merely accompany acinar injury but likely orchestrates a specific gene regulatory network that couples mitochondrial stress sensing to the activation of the Snx9-dependent vesicle export machinery.

### Runx1 Enhances AP-Induced Formation of mtDNA-Containing Ex-MDVs in PACs

**Fig. 2:**
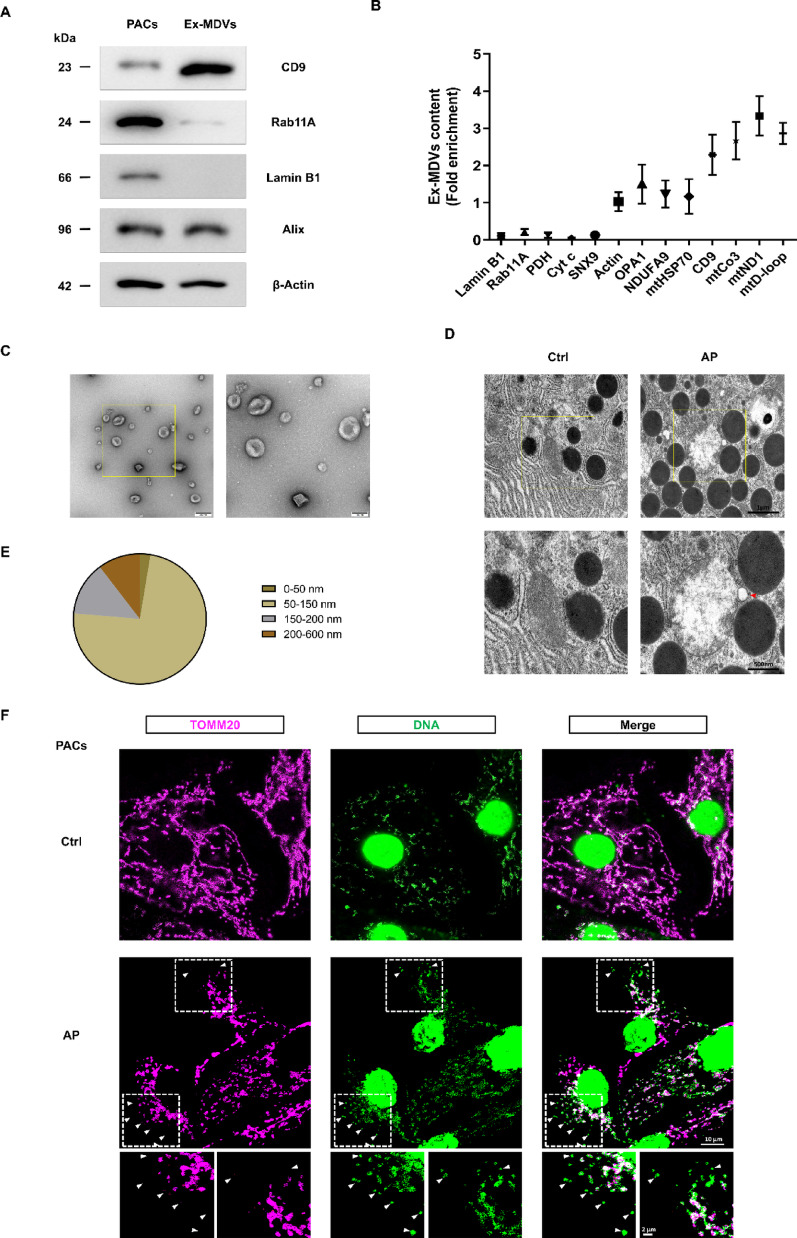
Characterization of extracellular mitochondrial-derived vesicles (Ex-MDVs) from PACs. (**A**) Western blot analysis of Ex-MDV markers (CD9, Alix), Rab11a, Lamin B1, and β-Actin. (**B**) Quantification of mitochondrial protein enrichment in Ex-MDVs. (**C**) TEM image of PAC-derived extracellular Ex-MDVs. (**D**) TEM images of mitochondria in PACs showing intracellular budding vesicles (MDVs) in AP conditions. (**E**) Size distribution of Ex-MDVs. (**F**) Immunofluorescence of TOM20 and DNA

To determine whether the transcriptional activation of vesicle transport corresponds to mitochondrial vesicle release, we examined extracellular vesicles derived from PACs. Western blotting confirmed the enrichment of EV markers (CD9 and Alix) in PAC-derived Ex-MDVs. Notably, Rab11A was abundant in PACs lysates but absent in Ex-MDVs, consistent with its role as a regulator of intracellular trafficking rather than a cargo protein. The nuclear marker Lamin B1 was undetectable (Fig. 2A). Quantitative proteomics demonstrated significant enrichment of mitochondrial proteins (mt-Co3 and mt-ND1) in AP-derived Ex-MDVs (Fig. 2B), suggesting selective packaging of mitochondrial components.

TEM revealed intact, membrane-bound vesicles in the extracellular space (Fig. 2C), Crucially, ultrastructural examination of the intracellular compartment revealed disrupted mitochondria exhibiting distinct budding events in AP PACs (Fig. 2D). These structures resemble MDVs, representing the initial biogenesis step prior to extracellular release. In contrast, such budding events were absent in controls. Particle size analysis showed that the majority of PAC-derived Ex-MDVs ranged from 50 to 150 nm in size (Fig. 2E). To further characterize the isolated Ex-MDVs, nanoparticle tracking analysis (NTA) was performed. The peak analysis showed a single dominant peak centered at 120.6 nm, accounting for 100% of the detected peak population under the present measurement conditions, without obvious secondary peaks. The measured particle concentration was 6.6 × 10^7^ particles/mL after dilution, corresponding to an original concentration of 2.0 × 10^11^ particles/mL (Fig. S7). These results further support the purity, concentration, and nanosized distribution of the Ex-MDVs preparation. Immunofluorescence staining confirmed mitochondrial fragmentation and extranuclear DNA signals in AP PACs, contrasting with the preserved mitochondrial networks in controls (Fig. 2F). To evaluate extracellular mtDNA under AP conditions, we assessed LDH release and separately quantified mtDNA in the PACs supernatant and PACs-derived EVs under AP conditions. In AP, LDH was significantly increased, indicating the loss of PACs membrane integrity and subsequent cell death (Fig. S4A). Consistent with this, free mtDNA levels in the culture supernatant were significantly elevated (Fig. S4B). Importantly, mtDNA abundance was also markedly increased in the PACs EVs (Fig. S4C), indicating that AP was associated with increased mtDNA levels in PAC-derived EVs. These findings support the presence of mtDNA within Ex-MDVs under AP conditions.

Since AP induced the release of mtDNA-containing Ex-MDVs, we next examined whether Runx1 functionally contributes to this process. We established an overexpression system in PACs and collected the Ex-MDVs (Fig. S1A-C). Runx1 overexpression markedly increased mitochondrial reactive oxygen species (mtROS) production, which was further elevated upon AP induction (Fig. S1D). Moreover, qPCR analysis revealed that Ex-MDVs released from Runx1-overexpressing PACs contained significantly higher levels of mtDNA *(mtCo3*, *mtND1*, and *mtDloop*), with the greatest enrichment observed under combined Runx1 overexpression and AP induction conditions (Fig. S1E-G). These findings establish that Runx1 enhances mitochondrial stress and promotes the packaging of mtDNA into Ex-MDVs.

### Runx1 Binds Directly to the *Snx9* promoter to Initiate Its Transcriptional Activation

**Fig. 3:**
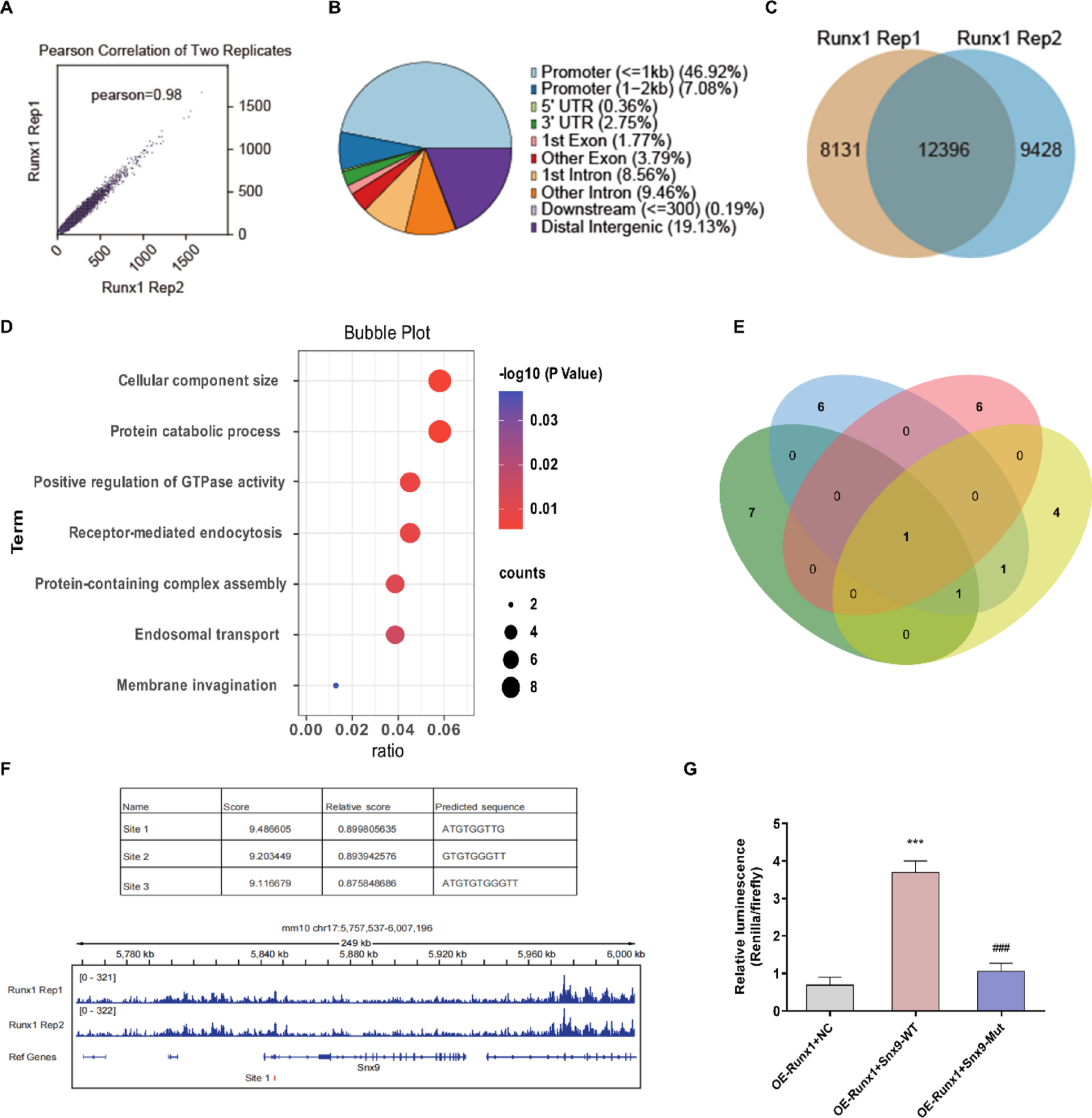
CUT&Tag analysis revealed Runx1 as a direct transcriptional activator of the *Snx9* promoter. (**A**) Correlation analysis of biological replicates showing high reproducibility of the Runx1 CUT&Tag peaks (Pearson *r* = 0.98). (**B**) Genomic distribution of Runx1 binding sites, showing preferential occupancy at promoter regions. (**C**) Venn diagram showing the overlap of consistent Runx1 peaks between replicates. (**D**) Bubble plot of Gene Ontology (GO) enrichment analysis for Runx1 target genes, highlighting pathways related to vesicle transport and cellular component organization. **(E**) Venn diagram illustrating the intersection of Runx1-bound genes with the enriched GO categories identified in (**D**). (**F**) Visualization of the *Snx9* genomic locus showing specific Runx1 binding peaks at the promoter region, alongside predicted binding motifs. (**G**) Luciferase reporter assay validating Runx1-dependent transcriptional activation of the *Snx9* promoter. PACs were co-transfected with Runx1 overexpression vector (OE-*Runx1*) and either wild-type (WT) or mutant (Mut) *Snx9* promoter constructs. NC: negative control (empty vector). Data are presented as the mean ± SD. ^***^p < 0.001 vs. OE-Runx1 + NC; ^###^*p* < 0.001 vs. OE-Runx1 + Snx9-WT

Given the strong correlation between Runx1 and Snx9 expression in AP PACs, we investigated their direct interaction. CUT&Tag analysis demonstrated high reproducibility between biological replicates (Fig. 3A) and identified genome-wide Runx1 binding sites predominantly located in promoter regions (Fig. 3B). A total of 12,936 common peaks were detected across replicates (Fig. 3C). Functional enrichment analysis revealed that Runx1 binding sites were associated with GO categories including receptor-mediated endocytosis and endosomal transport (Fig. 3D). Venn diagram analysis confirmed the overlap of Runx1 binding peaks with genes in these categories (Fig. 3E). Importantly, binding motif prediction and visualization of the *Snx9* locus confirmed Runx1 occupancy within its promoter region (Fig. 3F). Luciferase reporter assays validated that Runx1 expression significantly increased *Snx9* promoter activity, an effect abolished by mutation of the Runx1 binding site (Fig. 3G). Together, these results demonstrate that Runx1 directly activates *Snx9* transcription, providing a mechanistic link between Runx1 expression and the vesicle transport-dependent release of Ex-MDVs.

### Runx1-Snx9 signaling promotes mitochondrial stress and mtDNA release via MDVs

**Fig. 4:**
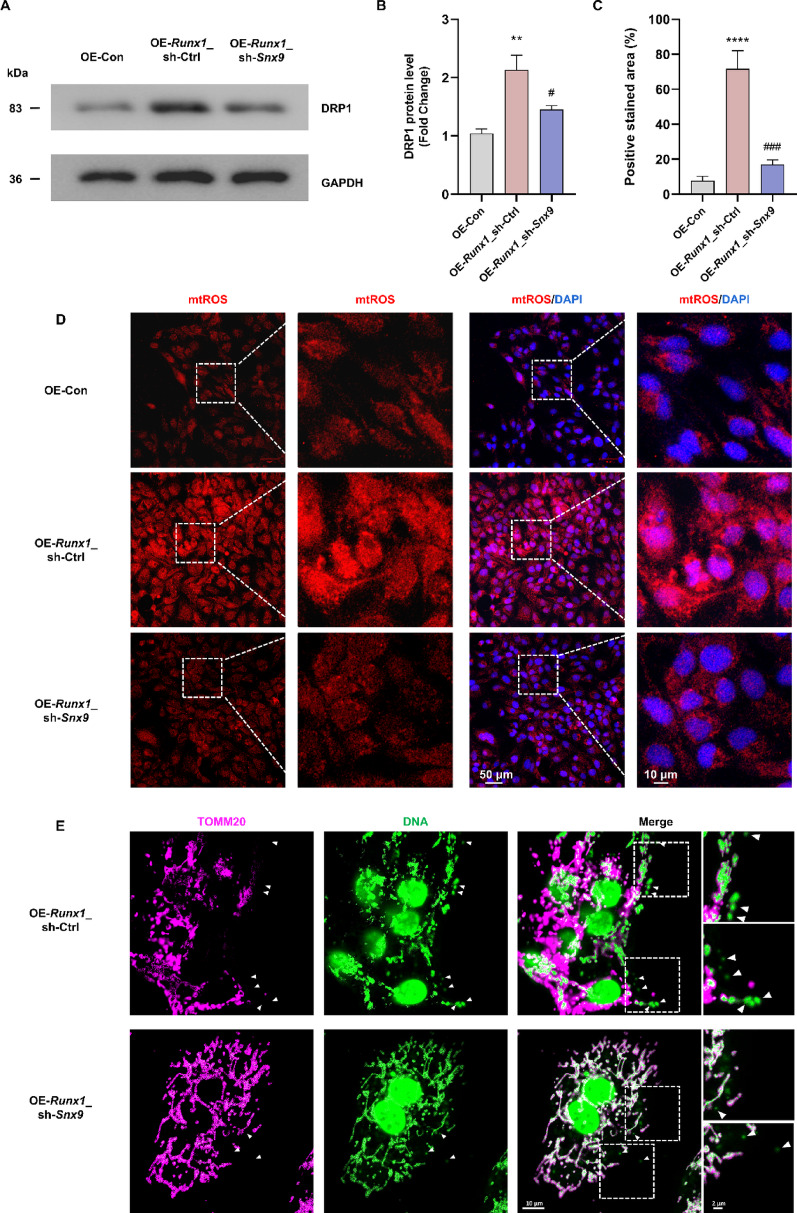
The Runx1-Snx9 axis drives Drp1-mediated mitochondrial fission and mtDNA leakage in PACs. (**A**) Western blot analysis of DRP1 protein levels in PACs following Runx1 overexpression (OE-*Runx1*) with or without Snx9 knockdown (sh-*Snx9*). GAPDH served as a loading control. (**B**) Densitometric quantification of DRP1 protein levels relative to GAPDH. (**C**,** D**) Quantification (**C**) and representative immunofluorescence images (**D**) of mitochondrial reactive oxygen species (mtROS, red) in PACs. Nuclei were counterstained with DAPI (blue). Note that Snx9 knockdown mitigates Runx1-induced oxidative stress. (**E**) Immunofluorescence staining of TOM20 (mitochondria, magenta) and DNA (green). Runx1 overexpression induces mitochondrial fragmentation and the accumulation of extranuclear DNA signals (indicative of MDV formation or cytosolic mtDNA), which is rescued by Snx9 silencing. Arrowheads indicate cytosolic mtDNA/MDV puncta

Because mitochondrial fission is a prerequisite for the generation of MDVs, we next examined whether Runx1-Snx9 signaling promotes the expression of Drp1, a key driver of mitochondrial fragmentation [4]. Western blot analysis revealed that the DRP1 protein levels markedly increased following Runx1 overexpression, whereas Snx9 knockdown substantially attenuated this effect (Fig. 4A, B). Immunofluorescence staining demonstrated increased levels of mtROS in PACs overexpressing Runx1, which was significantly diminished by Snx9 knockdown (Fig. 4C, D). Additionally, co-staining of TOM20 and DNA revealed mitochondrial fragmentation and aberrant mtDNA localization in Runx1-overexpressing PACs, phenotypes that were largely rescued by Snx9 silencing (Fig. 4E). These results suggest that Runx1 increases Drp1 expression to promote mitochondrial stress and facilitates Snx9-mediated mitochondrial budding (MDV formation). Consequently, this leads to mtDNA leakage via Ex-MDVs, thereby linking the transcriptional activation of vesicle transport pathways to mitochondrial dysfunction.

### Runx1 deletion alleviates AP-induced pancreatic injury and mitochondrial dysfunction in vivo

**Fig. 5:**
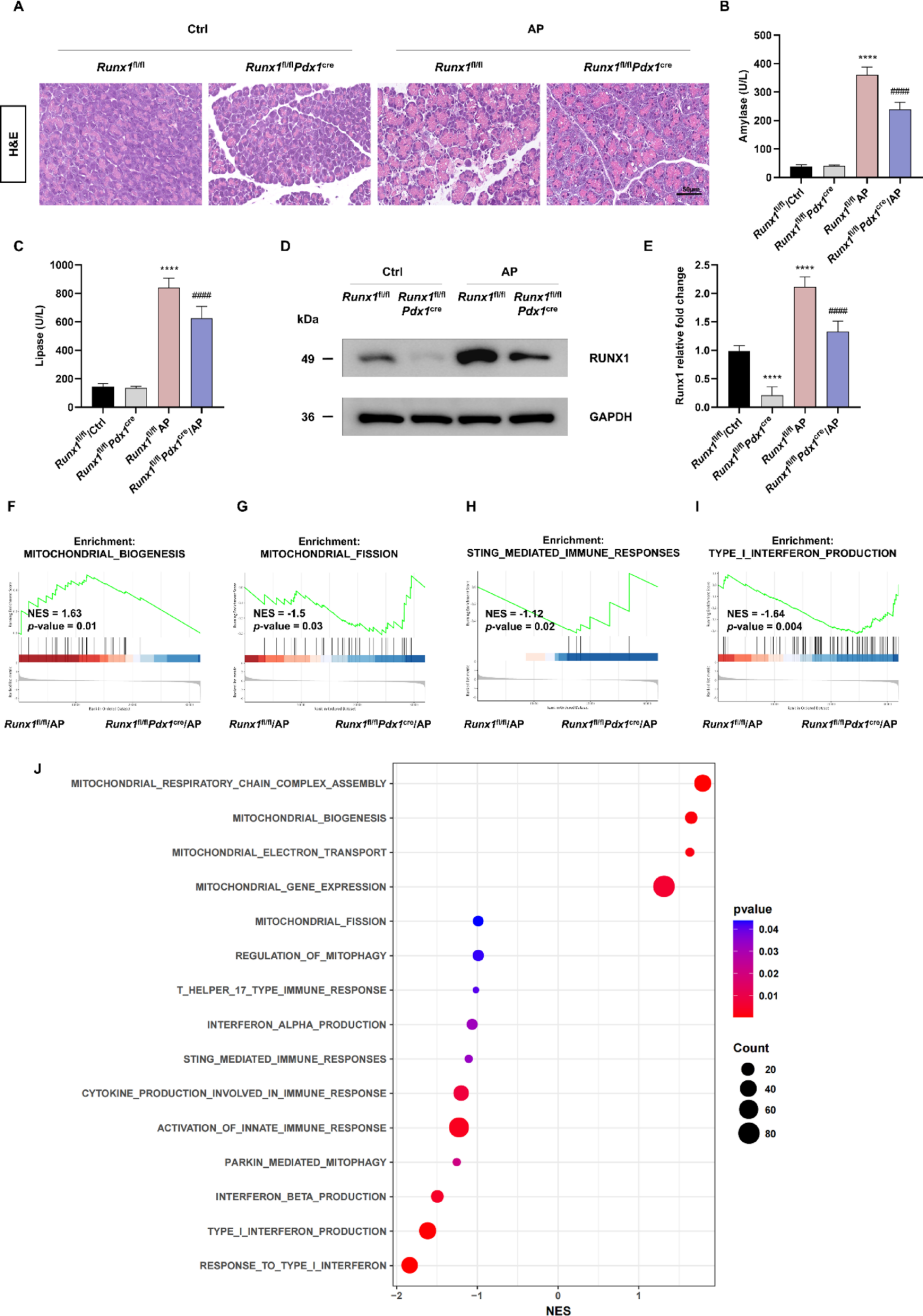
PAC-specific Runx1 deletion restores mitochondrial homeostasis and blunts STING-dependent immune responses in vivo. (**A**) Representative H&E staining of pancreatic tissue from control (Ctrl) and cerulein-induced AP mice. Runx1 deletion (Runx1^fl/fl^Pdx1^cre^) markedly reduces acinar necrosis and inflammatory infiltration compared to Runx1^fl/fl^ mice. Scale bar, 50 μm. (**B, C**) Serum amylase and lipase levels. (**D, E**) Western blot analysis and quantification of pancreatic RUNX1 expression, confirming efficient knockout. (**F–I**) GSEA enrichment plots from pancreatic tissue transcriptomics. Runx1 deletion suppresses mitochondrial fission and STING-mediated type I interferon production while restoring mitochondrial biogenesis pathways after AP induction. (**J**) Bubble plot summarizing GSEA results, highlighting the shift from pathological fission/inflammation to restorative biogenesis in knockout mice. Data are presented as the mean ± SEM. ^****^p < 0.0001 vs. Runx1^fl/fl^/Ctrl; ^####^p < 0.0001 vs. Runx1^fl/fl^/AP

To investigate the in vivo role of Runx1, we generated PAC-specific Runx1 knockout mice (*Runx1*^fl/fl^*Pdx1*^cre^) (Fig. S2A-C). Histological analysis revealed that cerulein-induced AP caused extensive pancreatic damage, including PAC necrosis and inflammatory infiltration, in *Runx1*^fl/fl^ mice, which was markedly attenuated in *Runx1*^fl/fl^*Pdx1*^cre^ mice (Fig. 5A). Consistently, serum amylase and lipase levels were reduced by Runx1 deletion (Fig. 5B, C). Western blot analysis confirmed that AP-induced Runx1 expression was abrogated in knockout mice (Fig. 5D, E).

Transcriptomic profiling followed by GSEA revealed that after AP induction, mitochondrial biogenesis was significantly enriched in *Runx1*^fl/fl^*Pdx1*^cre^ mice compared with controls, whereas mitochondrial fission was suppressed (Fig. 5F, G). In parallel, STING-mediated responses and type I interferon production were markedly suppressed upon Runx1 deletion (Fig. 5H, I). The GSEA bubble plot further supported the coordinated suppression of innate immune activation in Runx1-deficient mice (Fig. 5J). Together, these findings indicate that Runx1 deletion protects against AP-induced pancreatic injury by enhancing mitochondrial function while dampening innate immune activation.

### Runx1 Deficiency in PACs Suppresses AP-Induced Ex-MDVs Release and Dampens mtDNA-cGAS-STING Signaling

To functionally validate the transcriptomic predictions, we focused on the cGAS-STING pathway because of its role in sensing mtDNA-containing vesicles [5]. To distinguish the respective contributions of Runx1 activation and AP stimulation in the macrophage co-culture system, we included Ex-MDVs derived from non-AP PACs with or without Runx1 overexpression, as well as Ex-MDVs from AP-treated PACs with or without Runx1 overexpression. Ex-MDVs derived from Runx1-overexpressing PACs markedly increased cGAS and STING expression and IRF3 phosphorylation in BMDMs (Fig. S3A–I). Together, these data indicate that Runx1 and AP make distinct yet cooperative contributions to the proinflammatory activity of PAC-derived Ex-MDVs in macrophages.

**Fig. 6:**
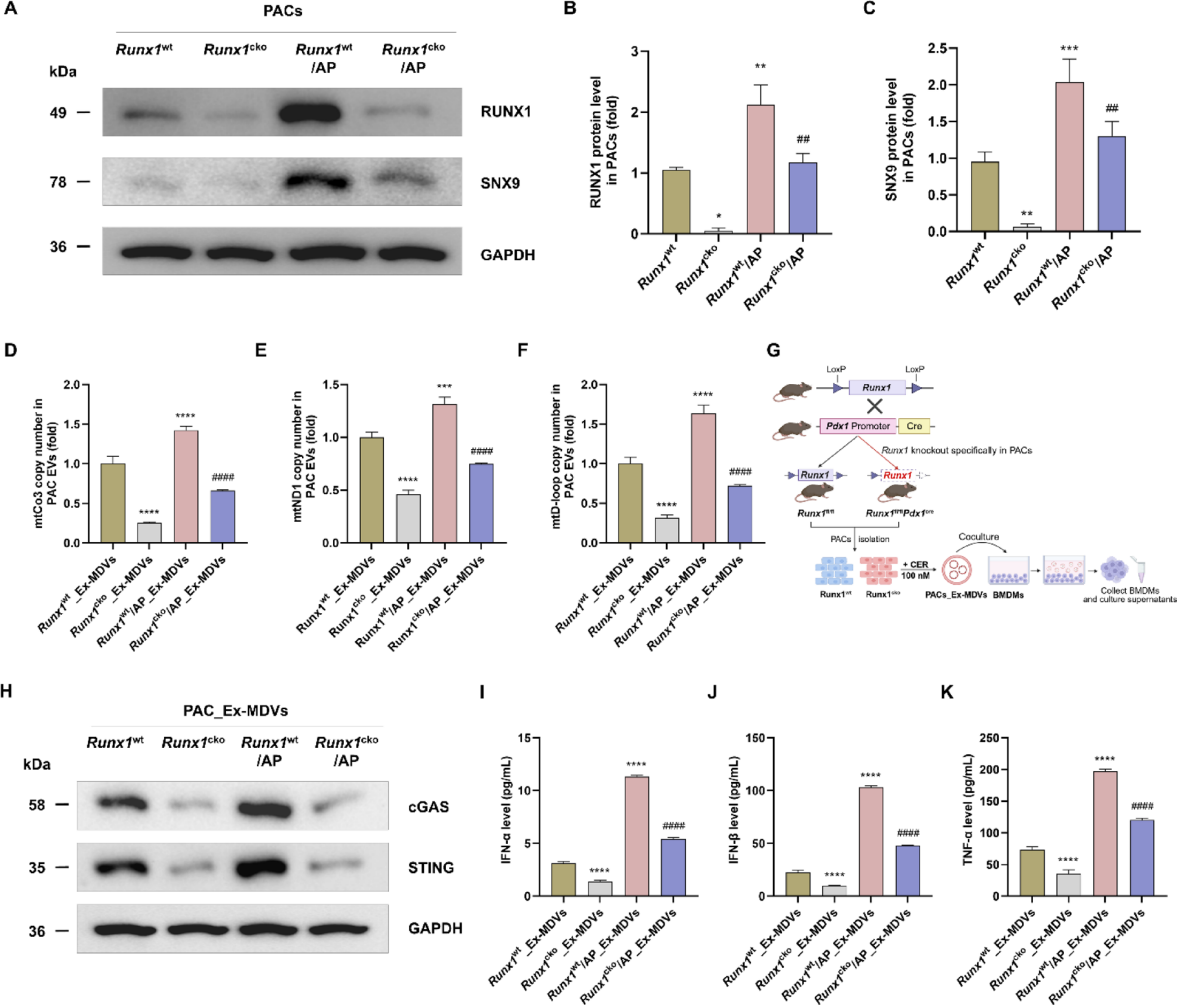
Runx1 deficiency prevents the secretion of pathogenic, mtDNA-enriched Ex-MDVs and abrogates macrophage cGAS–STING activation. (**A-C**) Western blot analysis (**A**) and quantification (**B**,** C**) of RUNX1 and SNX9 expression in primary PACs, confirming that Runx1 deletion prevents AP-induced Snx9 upregulation. (**D-F**) qPCR analysis of mitochondrial DNA cargo (*mt-Co3*, *mt-ND1*, *mt-Dloop*) within purified extracellular Ex-MDVs. Runx1 deletion significantly reduces the packaging of mtDNA into Ex-MDVs. (**G**) Schematic of the co-culture system: PAC-derived Ex-MDVs were treated to bone marrow-derived macrophages (BMDMs). (**H**) Western blot analysis of cGAS and STING activation in BMDMs. (**I-K**) ELISA quantification of proinflammatory cytokines (IFN-α, IFN-β, TNF-α) in BMDM supernatants. Ex-MDVs from Runx1-deficient PACs fail to trigger the cGAS–STING inflammatory cascade. Data are shown as the mean ± SEM. ^*^p < 0.05, ^**^p < 0.01, ^****^p < 0.0001, vs. *Runx1*^fl/fl^; ^##^*p* < 0.01, ^####^*p* < 0.0001, vs. *Runx1*^fl/fl^/AP

We next examined the loss-of-function model. Primary PACs from *Runx1*^fl/fl^ and *Runx1*^fl/fl^*Pdx1*^cre^ mice (*Runx1*^wt^ and *Runx1*^cko^) were stimulated to established the AP model. Western blotting confirmed that AP induction markedly increased RUNX1 and SNX9 expression in *Runx1*^wt^ PACs, whereas these effects were attenuated in *Runx1*^cko^ PACs (Fig. 6A–C). qPCR analysis revealed that AP-induced enrichment of *mt-Co3*, *mt-ND1*, and *mt-Dloop* in PAC-derived Ex-MDVs was significantly reduced upon Runx1 deletion (Fig. 6D–F). To further determine whether SNX9 acts downstream of RUNX1 in regulating Ex-MDVs export, we performed a rescue experiment by re-expressing Snx9 in Runx1-deficient PACs. Western blot analysis confirmed that SNX9 protein levels, which were markedly reduced by Runx1 silencing, were restored following Snx9 re-expression (Fig. S6A, B). Importantly, qPCR analysis showed that the decreased copy numbers of mtCo3, mtND1, and mtD-loop in PACs-derived Ex-MDVs caused by Runx1 silencing were significantly rescued by Snx9 re-expression (Fig. S6C-E). These findings support that SNX9, as the downstream target of RUNX1, is involved in the regulation of MDVs biogenesis and Ex-MDVs secretion. Functionally, Ex-MDVs from *Runx1*^wt^/AP PACs strongly activated the cGAS-STING pathway in BMDMs and induced cytokine secretion (IFN-α, IFN-β, TNF-α), phenomena that were markedly diminished by Ex-MDVs from *Runx1*^cko^/AP PACs (Fig. 6G–K). To determine whether this effect depended on Ex-MDVs-encapsulated mtDNA, purified AP-derived Ex-MDVs were pretreated with DNase I in the presence or absence of Triton X-100 before macrophage exposure. DNase I treatment alone did not impair AP_Ex-MDVs-induced cGAS and STING activation, whereas membrane disruption with Triton X-100 rendered the encapsulated mtDNA susceptible to DNase I digestion and abolished the stimulatory effect of AP_Ex-MDVs (Fig. S5A-C). Together, these results indicate that RUNX1 promotes the release of mtDNA-enriched Ex-MDVs from PACs through a SNX9-dependent mechanism, and that mtDNA-containing Ex-MDVs contributes to the activation of cGAS-STING signaling in macrophages.

### Runx1 Deletion and cGAS inhibition attenuate AP-Induced M1 Macrophage Activation In Vivo

**Fig. 7:**
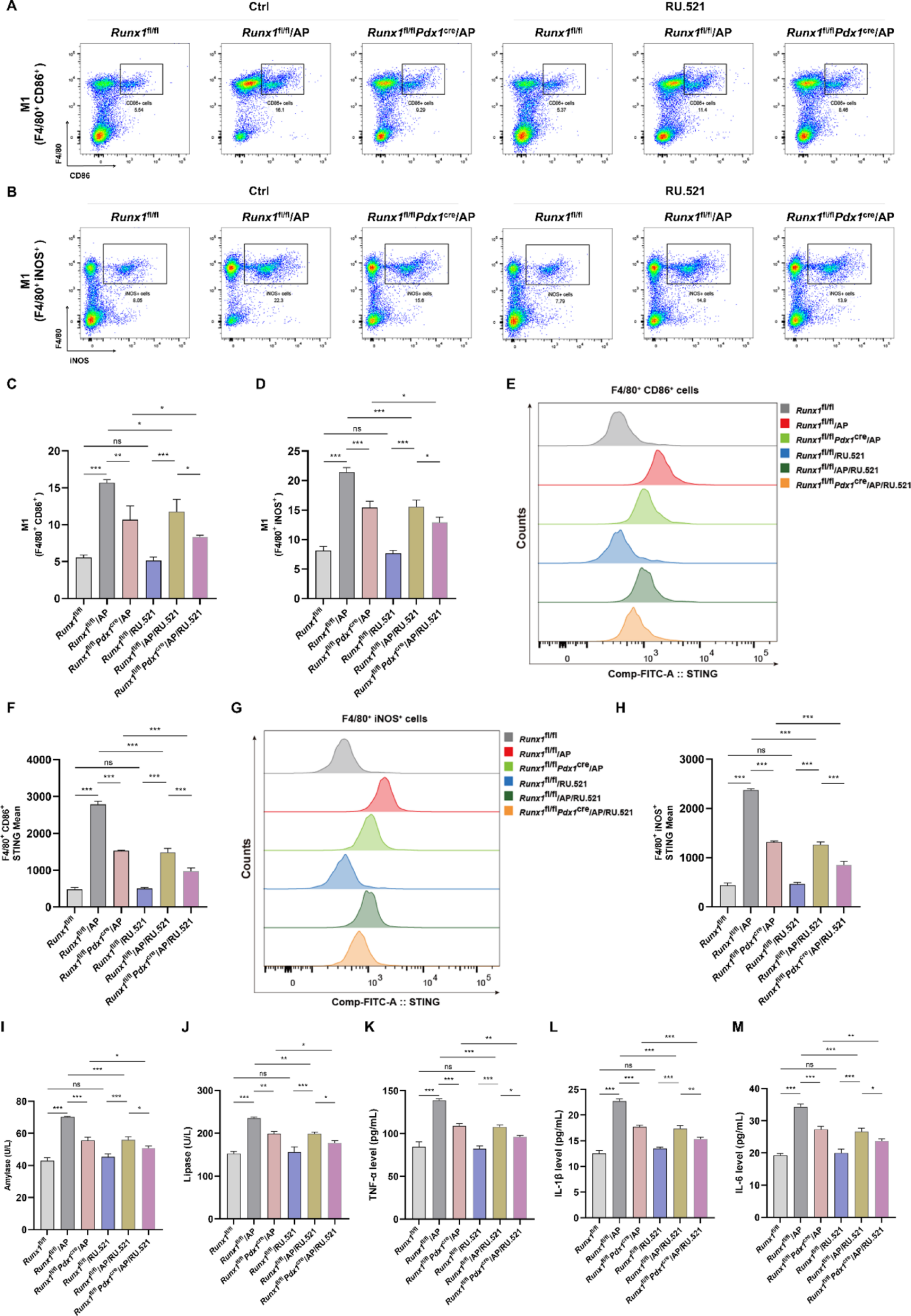
Blocking the Runx1-Ex-MDV-STING axis attenuates proinflammatory M1 macrophage polarization and systemic inflammation in vivo. (**A**,** B**) Flow cytometry plots of pancreatic immune cell infiltration, showing F4/80⁺CD86⁺ and F4/80⁺iNOS⁺ M1 macrophages. (**C**,** D**) Quantification of M1 macrophage subsets. (**E-H**) Representative histograms and quantification of STING expression within CD86⁺ (**E**,** F**) and iNOS⁺ (**G**,** H**) macrophage populations. Both Runx1 deletion in PACs and systemic cGAS inhibition (RU.521) blunt STING activation in infiltrating macrophages. (**I-M**) Analysis of systemic injury markers (amylase, lipase) and serum cytokines (TNF-α, IL-1β, IL-6). Data are shown as the mean ± SEM. ^*^p < 0.05, ^**^p < 0.01, ^***^p < 0.001, ns: not significant

Because sustained STING activation influences macrophage polarization [6], we evaluated whether Runx1-dependent Ex-MDV signaling promotes proinflammatory phenotypes. In the co-culture system, Ex-MDVs derived from Runx1-overexpressing PACs increased the proportions of F4/80⁺CD86⁺ and F4/80⁺iNOS⁺ macrophages (Fig. S3G–J). Consistent with in vitro results, pancreatic tissue analysis revealed that the infiltration of F4/80⁺CD86⁺ and F4/80^+^iNOS^+^ M1 macrophages markedly increased in AP mice, which was significantly attenuated in *Runx1*^fl/fl^*Pdx1*^cre^/AP and cGAS inhibitor (RU.521)-treated mice (Fig. 7A–D). STING expression in M1 macrophage subsets was substantially upregulated in *Runx1*^fl/fl^/AP mice but decreased in Runx1-deficient and RU.521-treated mice (Fig. 7E–H). Furthermore, both Runx1 deletion and RU.521 treatment reduced serum amylase, lipase, and proinflammatory cytokine levels (Fig. 7I–M). These results indicate that Runx1 in PACs promotes macrophage activation and STING-mediated inflammatory signaling upon AP induction.

## Discussion

This study establishes the Runx1-Snx9 axis as a critical upstream orchestrator of Ex-MDV release from PACs during AP. By mechanistically linking transcriptional reprogramming to organelle dynamics and macrophage-driven hyperinflammation, our findings unveil a previously unrecognized pathway governing DAMP export. In this study, our experimental results support a model wherein Runx1 upregulation triggers a fundamental shift in mitochondrial fate: promoting the Snx9-dependent budding of intracellular MDVs, increases mtDNA loading into Ex-MDVs, and thereby enhances the release of mtDNA-enriched Ex-MDVs from PACs. This model summarizes the central findings supported by our data in the present study. This advances the current understanding of AP pathogenesis by shifting the paradigm from passive necrotic leakage to a transcriptionally regulated, active export of mtDNA.

The pathogenesis of AP is increasingly defined by a sterile systemic inflammatory response syndrome (SIRS) driven by DAMPs, with mtDNA emerging as a central mediator [15, 16]. Although traditionally viewed as a passive byproduct of lytic cell death, accumulating evidence suggests that mtDNA release involves regulated cellular processes [17]. In this study, we demonstrate that the Runx1-Snx9 transcriptional axis is essential for Ex-MDV generation. The induction of Runx1 during AP paralleled the upregulation of Snx9 and vesicle transport machinery. Functional assays confirmed that Runx1 does not merely correlate with injury but actively drives mitochondrial stress, fragmentation, Snx9-dependent MDV biogenesis, and the selective packaging of mtDNA into Ex-MDVs. These findings strongly argue that the systemic release of mtDNA in AP is a regulated cellular program, distinct from random necrosis.

Regarding the underlying biological mechanism, we identified Runx1 as a direct transcriptional activator of *Snx9* in PACs. Snx9 is a BAR-domain adaptor protein that coordinates membrane curvature and scission [18] and has been implicated in the biogenesis of MDVs [19, 20]. Under physiological conditions, MDVs shuttle oxidized mitochondrial cargo to lysosomes for degradation (micromitophagy), serving as a quality control mechanism to preserve the organelle network. However, our data suggest that in the context of AP, the Runx1-driven surge in Snx9 and Drp1 subverts this homeostatic machinery. Rather than being degraded, these mtDNA-laden MDVs may be preferentially handled by endosomal trafficking routes. Based on our single-cell transcriptomic analysis, we speculate that Rab11a-positive recycling endosomes or multivesicular bodies (MVBs) may participate in this process and thereby contribute to Ex-MDV secretion. This precise regulatory mechanism will be further verified in future experiments. The distinction between intracellular MDV formation and extracellular Ex-MDV release is critical for understanding AP pathology. While ruptured mitochondria release naked mtDNA that is susceptible to degradation by extracellular DNases, our findings suggest that vesicle trafficking provides a membrane-encapsulated, protected vehicle for mtDNA. Once delivered to macrophages via Ex-MDVs, this concentrated mtDNA payload potently activates the cytosolic cGAS-STING surveillance pathway. By delineating a Runx1-Snx9 axis that couples MDV biogenesis to aberrant exocytosis, our study reveals a sophisticated, transcriptionally controlled checkpoint that links local organelle stress to distant macrophage-mediated hyperinflammation.

Having established this axis as the driver of Ex-MDV release, we demonstrated its systemic consequences. PAC-derived Ex-MDVs acted as potent agonists of the cGAS-STING-IRF3 axis in macrophages, orchestrating an antiviral-like inflammatory response characterized by type I interferon production [21]. This intercellular crosstalk amplifies the inflammatory cascade far beyond the pancreas. While our functional validation focused on M1 macrophages, the implications likely extend to other immune compartments. Ex-MDVs may modulate neutrophils, dendritic cells, and notably B cells, which recent studies have identified as critical determinants of AP severity [22]. Our findings suggest that Ex-MDVs could serve as the “danger signal” that bridges acinar injury to the adaptive immune dysregulation observed in severe AP. Future studies are warranted to define the cell-type-specific uptake of these vesicles and their broad impact on the immune landscape.

These mechanistic insights highlight specific therapeutic opportunities. Runx1 inhibitors (e.g., Ro5-3335, Ro24-7429) have shown anti-inflammatory potential in other contexts [23, 24], and silencing Runx1 reduces immune infiltration in liver models [25]. While Runx1 is often studied in the context of myeloid differentiation [26, 27], our findings underscore its pathogenic role within the parenchymal tissue (PACs), where it initiates DAMP release at the source. Thus, targeting Runx1 or Snx9 in PACs offers a strategy to block the biogenesis of pathogenic vesicles before they engage the immune system, potentially suppressing the systemic inflammatory storm at its inception.

Despite providing new mechanistic insights, several limitations should be acknowledged. First, our analysis of effector cells focused primarily on macrophages; however, the complex immune microenvironment of AP involves interplay with neutrophils and adaptive immune populations [22, 28], whose interactions with Ex-MDVs require further investigation. Second, while our PAC-specific knockout models confirm the role of acinar Runx1, we cannot exclude the contribution of Runx1 in immune cells to the overall phenotype. Third, although our data support a model where the Runx1-Snx9 axis enhances Drp1-mediated fission and MDV formation, the precise molecular machinery governing the fusion of these MDVs with the plasma membrane (exocytosis) warrants detailed characterization. Fourth, our mechanistic findings are based on murine models and primary cells; validating the Runx1-Snx9-Ex-MDV signature in human clinical samples remains a priority.

In conclusion, this study connects mitochondrial dysfunction in PACs to macrophage-driven hyperinflammation via a novel vesicle trafficking pathway. By highlighting the transcriptional regulation of vesicle transport as a driver of immune activation, our findings provide new insight into how local organelle stress escalates to systemic injury. These results support a model in which mtDNA release is not a passive consequence of necrosis but is facilitated by an actively regulated program involving the generation of MDVs and their pathological secretion as Ex-MDVs. Future studies should explore the broader molecular interplay between trafficking proteins and mitochondrial dynamics and assess the clinical utility of targeting this axis to treat severe acute pancreatitis.

## Supplementary Information

Below is the link to the electronic supplementary material.

Supplementary material 1.

Supplementary material 2.

## Data Availability

All the data generated or analyzed during this study are included in this published article and its supplementary information files.
