# Supplementary material for: Runx1-Snx9 axis drives the pathological secretion of mitochondrial-derived vesicles to activate cGAS-STING signaling in acute pancreatitis
Source: J Nanobiotechnology. 2026 Jun 16;24:671. doi: 10.1186/s12951-026-04687-6 (PMC13377763; doi:10.1186/s12951-026-04687-6)
Supplement: Supplementary file 2 — Supplementary material 2. [file 12951_2026_4687_MOESM2_ESM.docx]

**
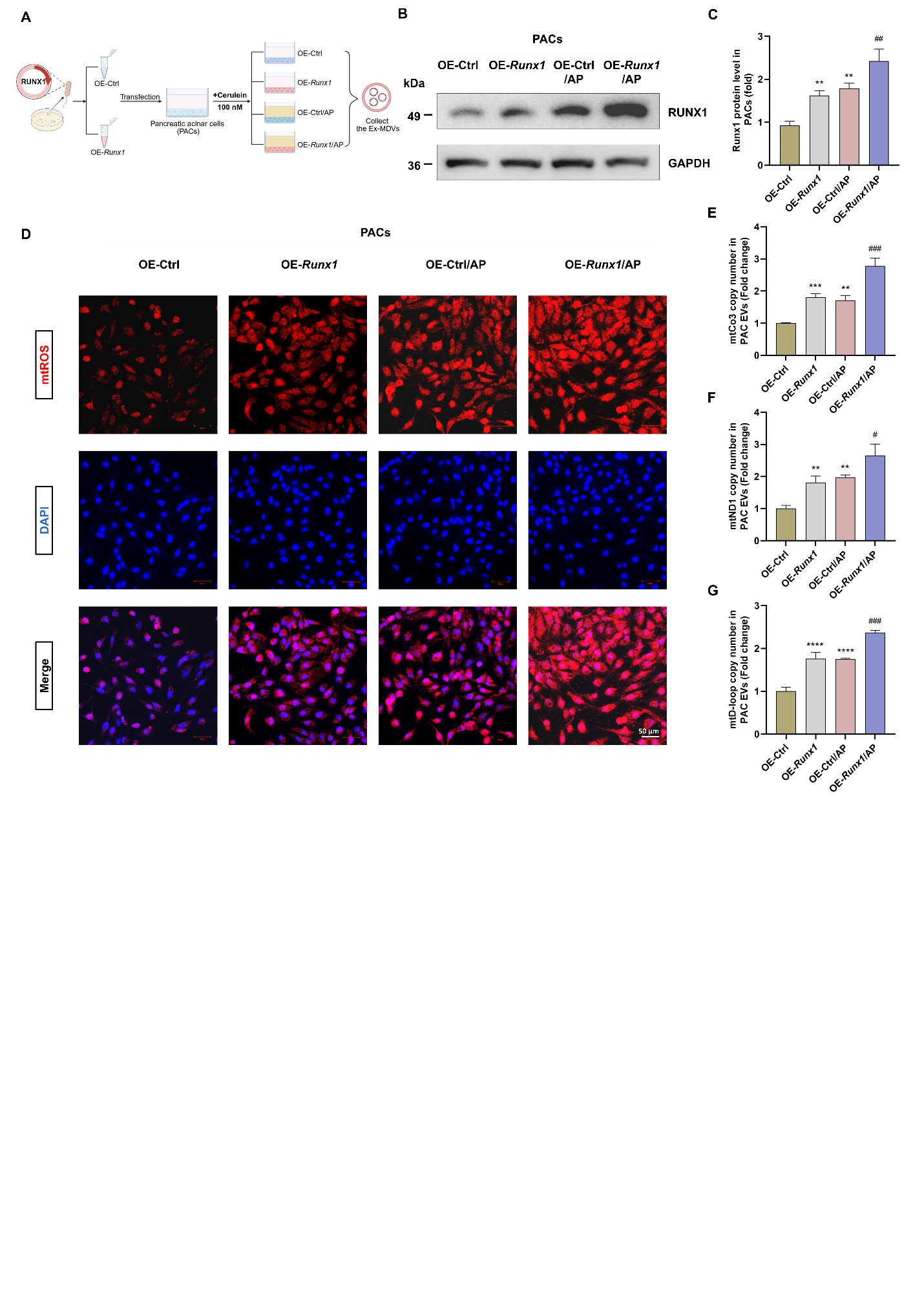
**

**Figure S1. Runx1 gain-of-function exacerbates mitochondrial stress and drives the selective packaging of mtDNA into Ex-MDVs.** (A) Schematic of the experimental design for Runx1 overexpression (OE) in PACs and Ex-MDV isolation. (B, C) Western blot validation of RUNX1 overexpression. (D) Immunofluorescence of mtROS (red) in PACs, showing that Runx1 overexpression amplifies oxidative stress under AP conditions. (E-G) qPCR quantification of mtDNA copy numbers (*mtCo3*, *mtND1*, *mtDloop*) in purified Ex-MDVs. Runx1 overexpression promotes the active enrichment of mtDNA cargo into extracellular vesicles. Data are presented as the mean ± SEM. ***p* < 0.01, ****p* < 0.001, *****p* < 0.0001 vs. OE-Ctrl; ^#^*p* < 0.05, ^##^*p* < 0.01, ^###^*p* < 0.001 vs. OE-Ctrl/AP.

**
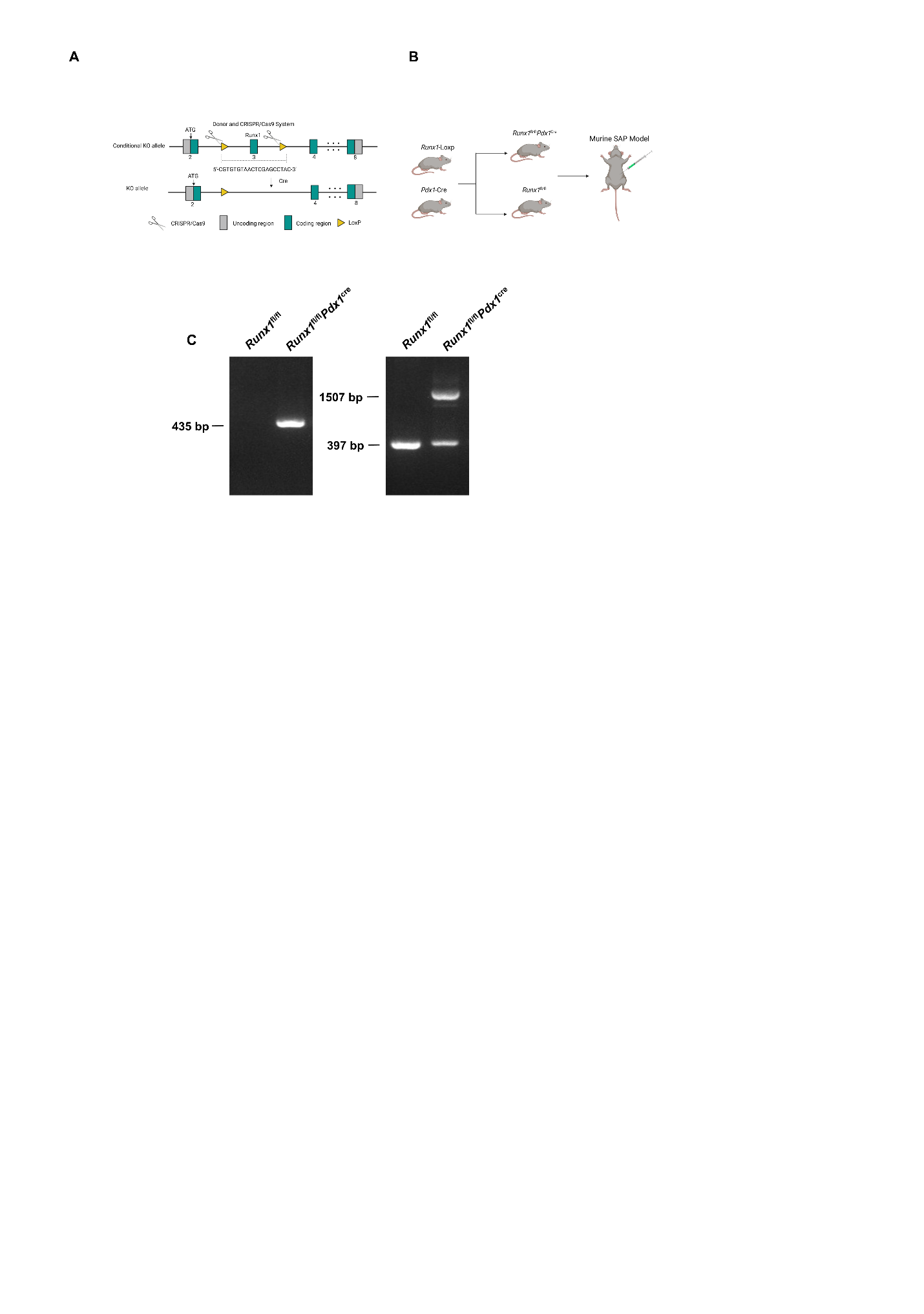
**

**Figure S2. Generation and validation of PAC-specific *Runx1* knockout mice.** (A) Schematic of the Runx1 conditional allele and CRISPR/Cas9 targeting strategy. (B) Breeding strategy to generate *Runx1*^fl/fl^*Pdx1*^cre^ mice. (C) PCR genotyping results confirming specific band sizes for *Runx1*^fl/fl^ and *Runx1*^fl/fl^*Pdx1*^cre^ alleles.

**
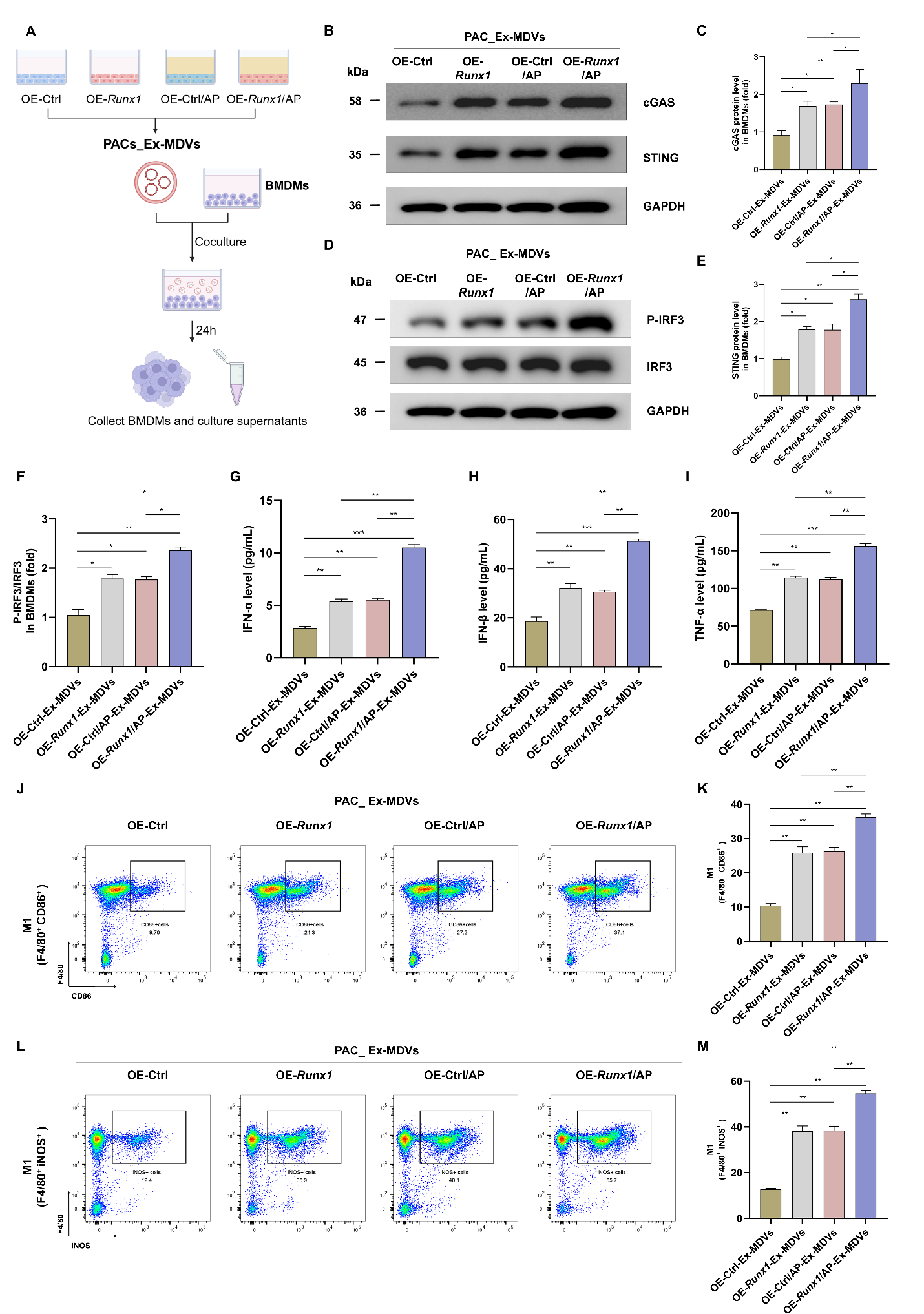
**

**Figure S3. Ex-MDVs from Runx1-overexpressing PACs serve as potent agonists for the macrophage cGAS-STING pathway.** (A) Schematic of the co-culture setup: transferring Ex-MDVs from OE-*Runx1* PACs to BMDMs. (B-F) Western blotting and quantification of the cGAS-STING signaling axis (cGAS, STING, IRF3, p-IRF3) in BMDMs. (G-I) ELISA quantification of cytokine release. (J-M) Flow cytometry analysis of M1 macrophage polarization (F4/80⁺CD86⁺ and F4/80⁺iNOS⁺). Runx1-driven Ex-MDVs significantly enhance macrophage inflammatory responses. Data are presented as the mean ± SEM; ^*^*p* < 0.05, ^**^*p* < 0.01, ^***^*p* < 0.001.


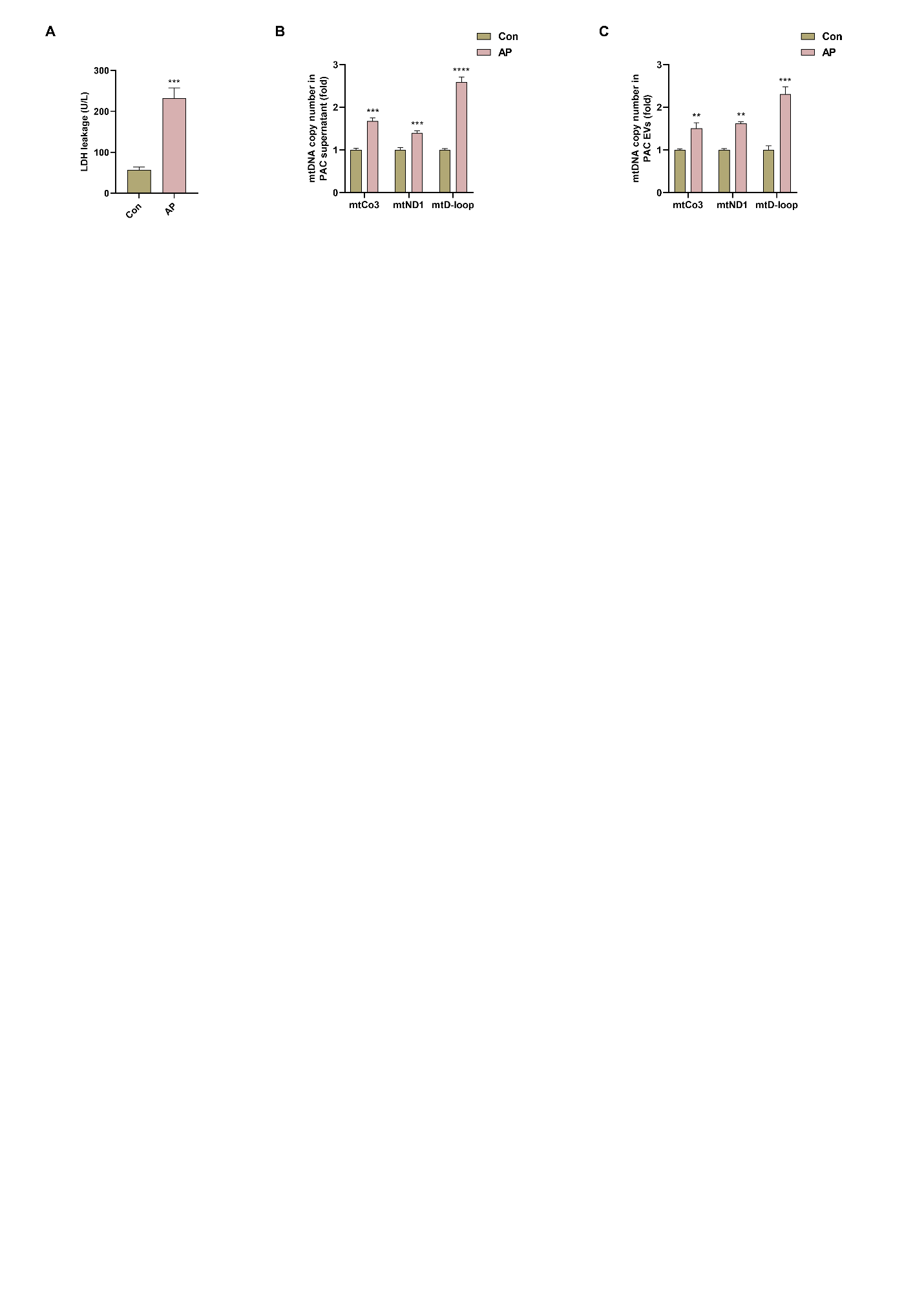


**Figure S4. AP promotes both cell death-associated free mtDNA release and Ex-MDVs-mediated mtDNA release from PACs.** (A) LDH leakage in the culture supernatant of control and AP-stimulated PACs. (B) qPCR analysis of mtDNA copy numbers (mtCo3, mtND1, and mtD-loop) in the PACs supernatant from control and AP groups. (C) qPCR analysis of mtDNA copy numbers in PACs-derived EVs from control and AP groups. Data are shown as the mean ± SD. **p < 0.01, ***p < 0.001, ****p < 0.0001.


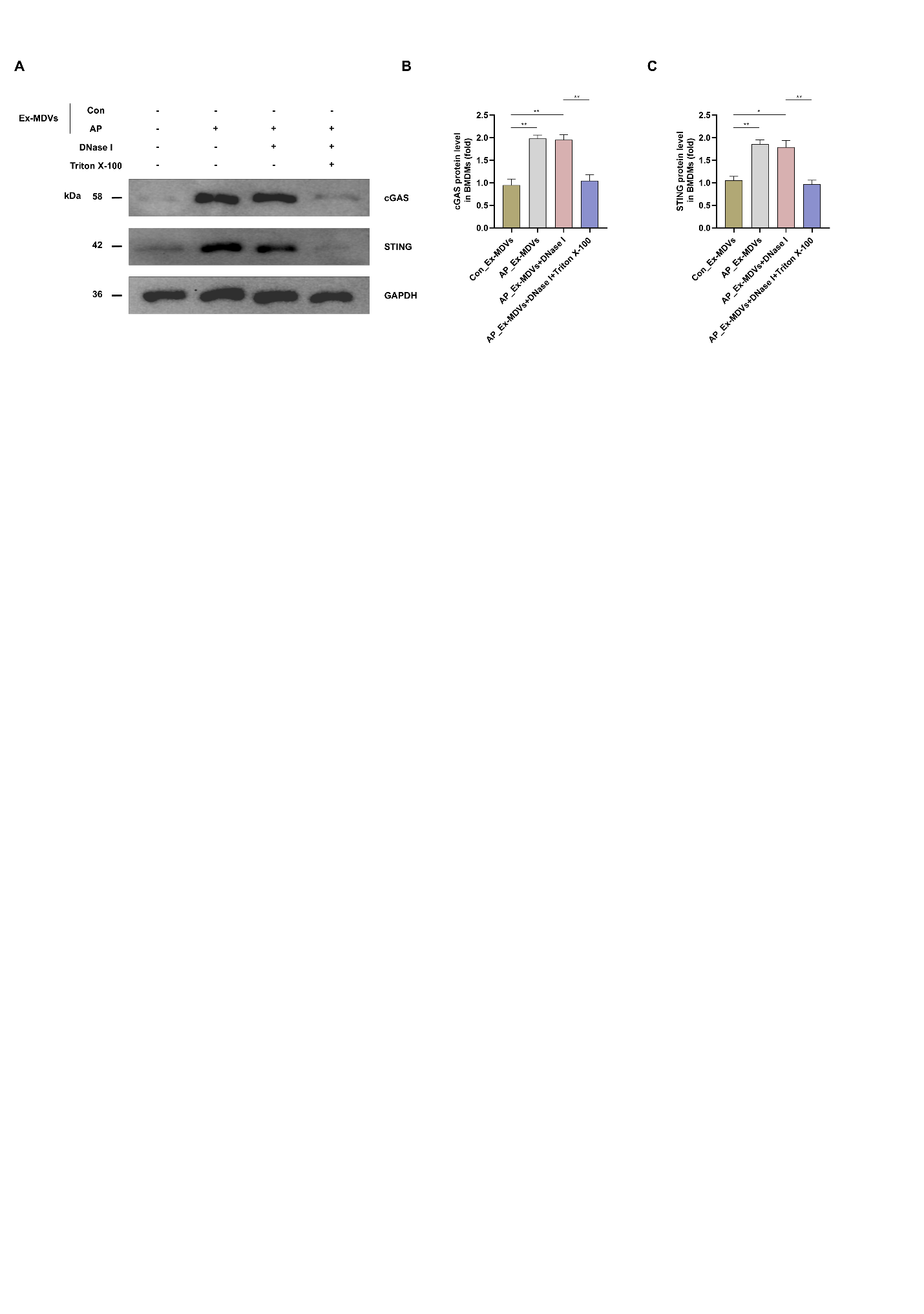


**Figure S5. Ex-MDVs are required for mtDNA-mediated activation of the cGAS-STING pathway in macrophages.** (A) Western blot analysis of cGAS and STING protein levels in macrophages treated with Ex-MDVs derived from control or AP PACs. AP_Ex-MDVs treated with DNase I in the presence or absence of Triton X-100. GAPDH was used as a loading control. (B, C) Quantification of cGAS (B) and STING (C) protein levels. Data are shown as the mean ± SD. *p < 0.05, **p < 0.01.


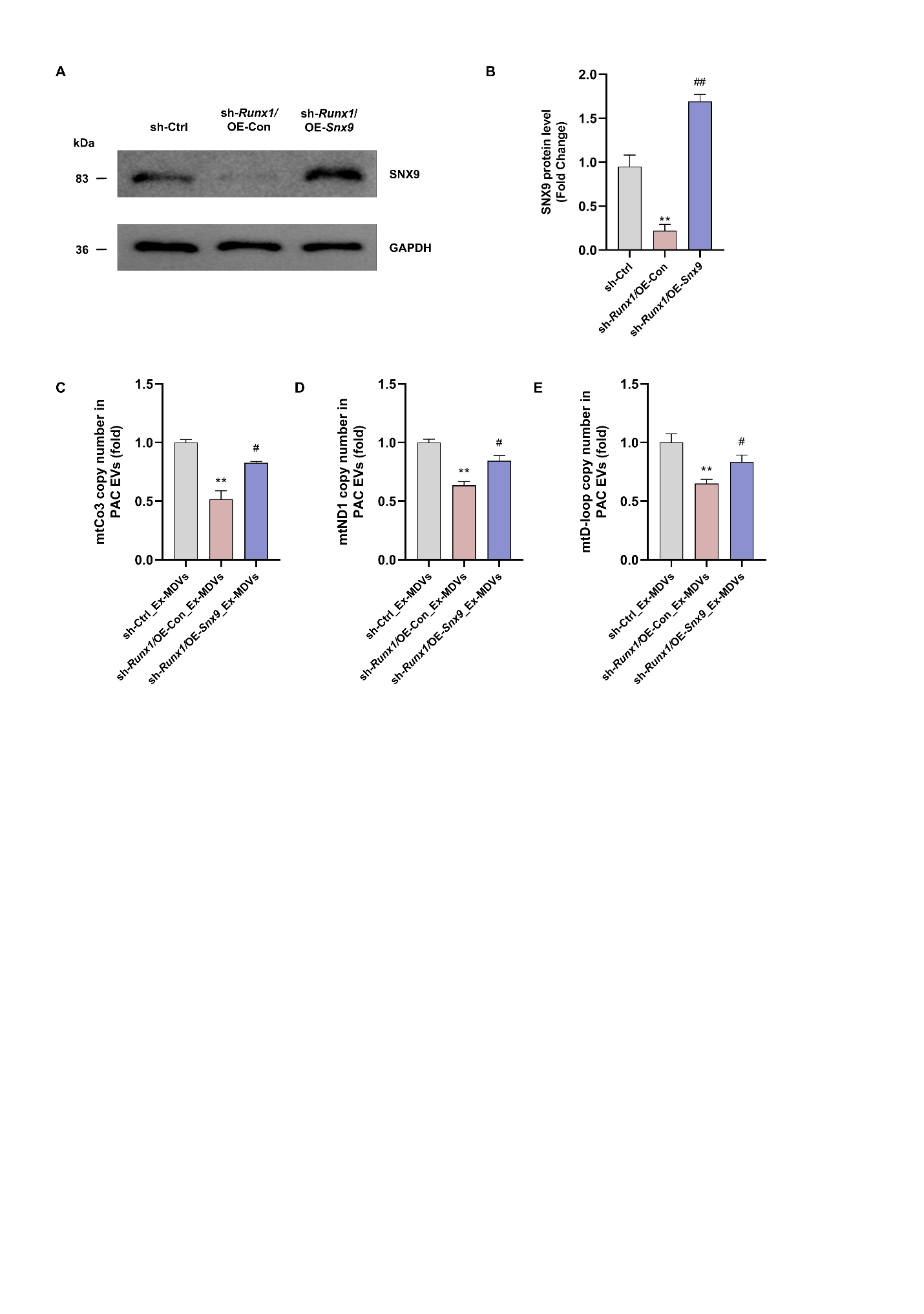


**Figure S6. Re-expression of Snx9 rescues the reduction of mtDNA cargo in Ex-MDVs caused by Runx1 silencing.** (A) Western blot analysis of SNX9 protein levels in PACs transfected with sh-Ctrl, sh-Runx1/OE-Con, or sh-Runx1/OE-Snx9. (B) Quantification of SNX9 protein levels. (C-E) qPCR analysis of mtCo3 (C), mtND1 (D), and mtD-loop (E) copy numbers in Ex-MDVs derived from the indicated PACs. Data are shown as the mean ± SD. **p < 0.01 vs. sh-Ctrl_Ex-MDVs; #p < 0.05, ##p < 0.01 vs. sh-Runx1/OE-Con_ Ex-MDVs.


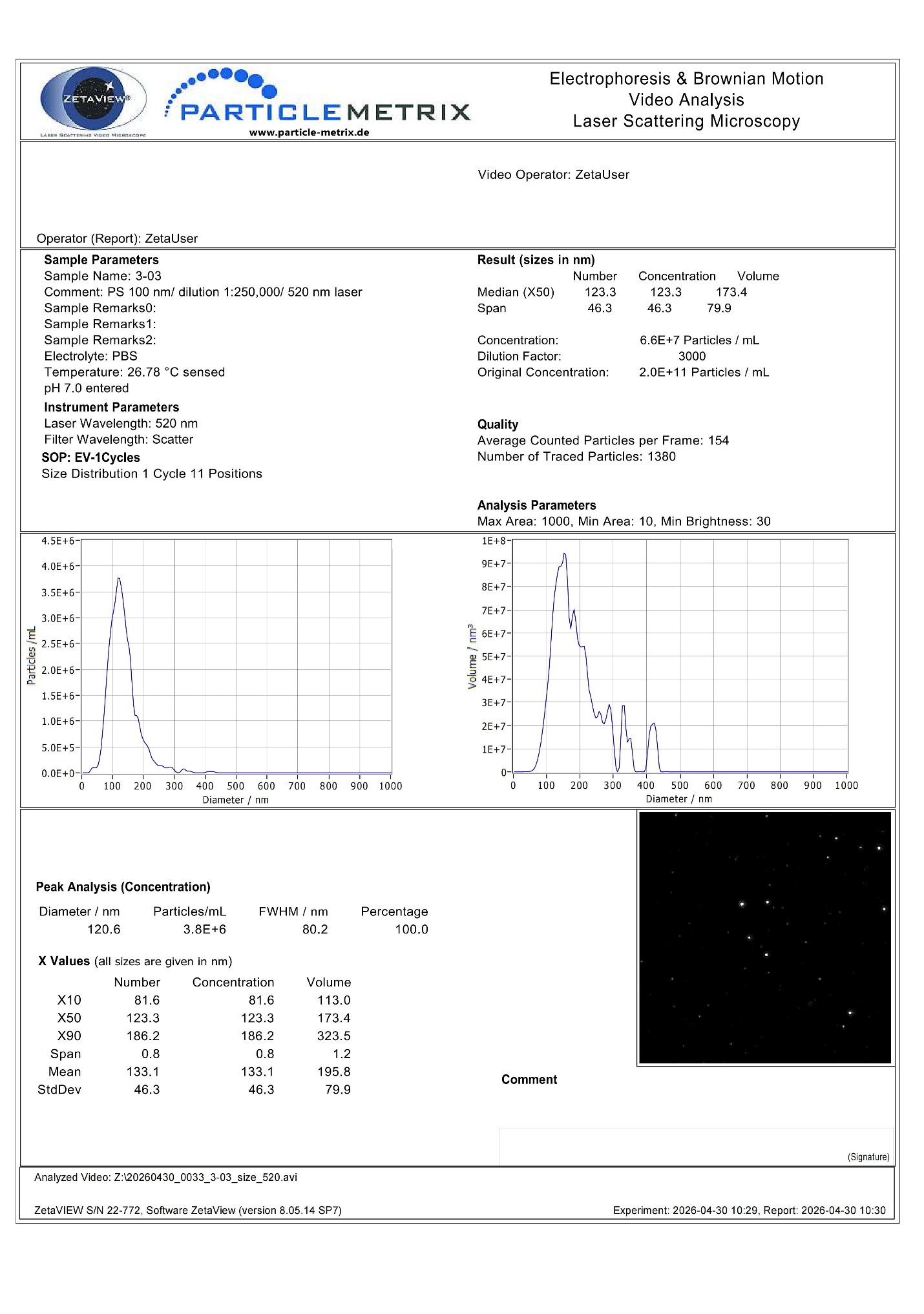


**Figure S7.** Nanoparticle tracking analysis (NTA) of purified Ex-MDVs.
